# Semi-Synthesis of Labeled Proteins for Spectroscopic Applications

**DOI:** 10.3390/molecules18010440

**Published:** 2013-01-02

**Authors:** Lucia De Rosa, Anna Russomanno, Alessandra Romanelli, Luca Domenico D’Andrea

**Affiliations:** 1 Istituto di Biostrutture e Bioimmagini, CNR, Via Mezzocannone 16, Napoli 80134, Italy; E-Mails: lucia.derosa@unina.it (L.D.R.); anna.russomanno@gmail.com (A.R.); 2 Dipartimento delle Scienze Biologiche, Università di Napoli “Federico II”, Via Mezzocannone 16, Napoli 80134, Italy; E-Mail: alessandra.romanelli@unina.it

**Keywords:** expressed protein ligation, intein, Förster resonance energy transfer, segmental labeling, NMR, protein labeling

## Abstract

Since the introduction of SPPS by Merrifield in the 60s, peptide chemists have considered the possibility of preparing large proteins. The introduction of native chemical ligation in the 90s and then of expressed protein ligation have opened the way to the preparation of synthetic proteins without size limitations. This review focuses on semi-synthetic strategies useful to prepare proteins decorated with spectroscopic probes, like fluorescent labels and stable isotopes, and their biophysical applications. We show that expressed protein ligation, combining the advantages of organic chemistry with the easy and size limitless recombinant protein expression, is an excellent strategy for the chemical synthesis of labeled proteins, enabling a single protein to be functionalized at one or even more distinct positions with different probes.

## 1. Introduction

Protein structure, folding dynamics, function and interactions with other macromolecules can be widely explored and characterized by biophysical techniques such as fluorescence and NMR spectroscopies. Spectroscopic techniques rely tightly on protein labeling strategies by which the chemical structure of a protein is modified through the introduction of biophysical probes, such as fluorophores or isotopes. A wide collection of protein labeling approaches have been developed in recent years [1,2,3,4], leading to great discoveries and innovations. In particular, the introduction of native chemical ligation (NCL) methodologies for chemical synthesis of proteins marked a breakthrough in protein and peptide chemistry, with a strong impact on chemical biology and biophysical applications [5,6]. Here, we review the semi-synthetic strategies employed for the preparation of labeled proteins and their spectroscopic applications.

### 1.1. Total Synthesis of Proteins: SPPS and Chemical Ligation

**Figure 1:**
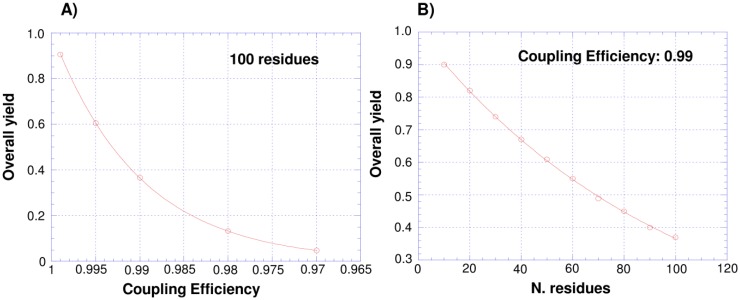
Dependence of stepwise SPPS yield on coupling efficiency (**A**) and number of coupling steps (**B**).

The great advantage of chemical protein synthesis and semi-synthesis over traditional recombinant protein expression refers to the precise control over the kind and number of modifications that can be introduced into a protein molecule, enabling one to realize any desired change of its covalent structure with surgical precision. Since the introduction in 1963 of the stepwise solid-phase peptide synthesis (SPPS) by Merrifield [7], chemical synthesis of proteins greatly expanded and culminated with the development of chemical ligation approaches [8]. Chemical ligation methods allow one to overcome the size-limit of SPPS, mainly due to incomplete coupling and deprotection reactions (Figure 1), affording the synthesis of large proteins by stitching together short synthetic segments to give a polypeptide chain of any desired length and harboring a plethora of possible chemical modifications.

**Figure 2:**
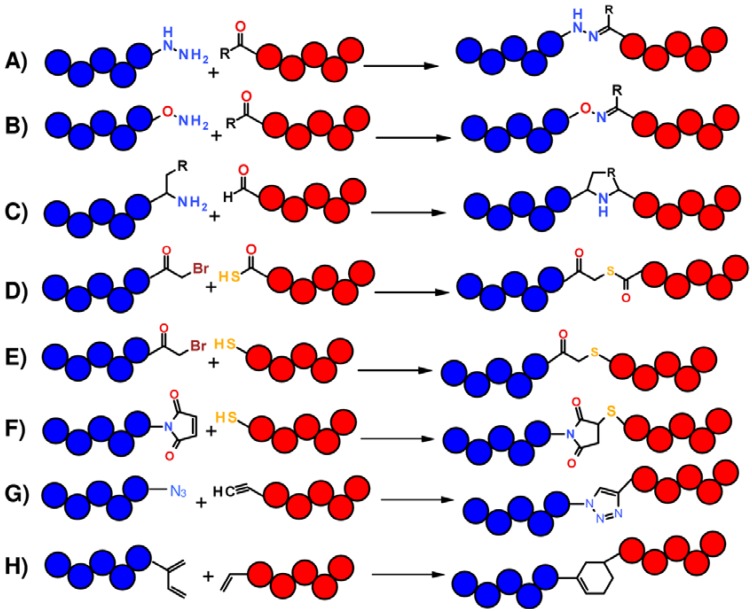
Schematic representation of chemoselective reactions useful for non-native chemical ligation. (**A**) and (**B**) R = H, CH_3_; (**C**) R = SH, OH.

In this scope, ligation strategies are based on chemoselective reactions between two functional groups respectively placed at N- and C-terminus of two contiguous peptide segments which react by forming a stable chemical bond. The type of chemical bond made at the site of junction between two peptide fragments depends on the reactive groups employed (Figure 2). For instance, the reaction between an hydrazide or an aminooxy group with an aldehyde or a ketone gives rise to hydrazones or oximes (Figure 2A,B); a pseudoproline analog results from the reaction between a peptide bearing a C-terminal glycolaldehyde ester and a second peptide starting with Cys, Ser or Thr [9,10] (Figure 2C); a peptide-thiocarboxylate reacts with a N^α^-bromoacetyl-peptide to give a thioester-linked polypeptide product (Figure 2D) [8]; the reaction between a thiol and a bromoacetyl group yields a polypeptide joined by thioether linkage (Figure 2E) [11]; thiol and maleimide groups react through a Michael addition (Figure 2F) [12]; Cu(I)-catalyzed azide-alkine [3+2] cycloaddition leads to the formation of a triazole ring (Figure 2G) [13,14]; Diels-Alder cycloaddition reaction, which involves a diene and a dienophile, yields a six member carbocycle (Figure 2H) [15].

**Figure 3:**
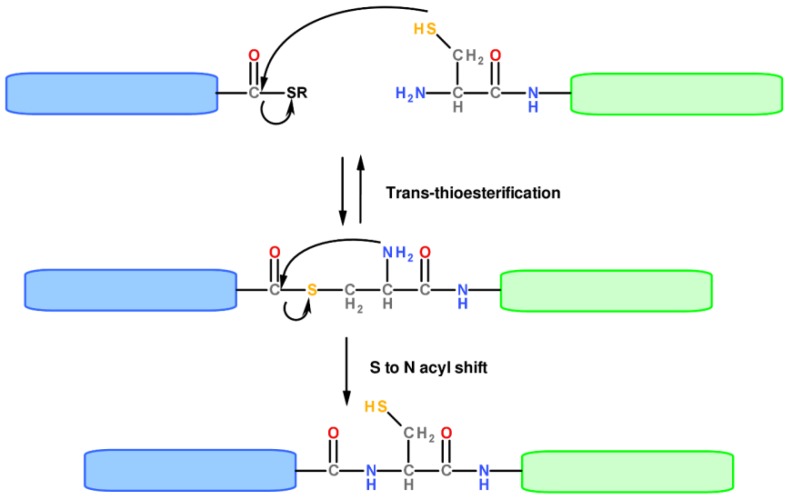
Mechanism of the NCL reaction.

Even though numerous proteins have been successfully prepared by chemical ligation strategies yielding a non-native bond at the junction sites [16,17], such unnatural structures are not always compatible with the assumption of correct protein folding. Thus, great efforts were directed towards the development of traceless chemical ligation approaches, ending up in the introduction of the NCL by Kent and coworkers [18]. This method leads to the formation of a single polypeptide chain bearing a native peptide bond at the ligation site after coupling of two peptide segments, one containing a carboxy-terminal α-thioester group and the other bearing a 1,2 aminothiol, like an amino-terminal Cys residue (Figure 3). The reaction occurs, even under mild conditions (aqueous solution at pH around neutrality), through a trans-thioesterification reaction which leads to the formation of a thioester intermediate rapidly evolving toward the desired stable amide-linked product through a spontaneousintramolecular S-to-N acyl shift. This reaction is orthogonal with respect to functional groups present in a protein and also proceeds selectively in the presence of internal Cys residues. Another powerful chemical ligation method is the traceless Staudinger ligation, which relies on the selective reaction between a phosphinothioester and an azide to form an amide bond [19]. This method does not necessitate of a Cys residue at the junction between two peptide fragments and, thus, appears as a strategy complementary to NCL.

The efficacy of the NCL has been significantly broadened by the introduction of a “recombinant” version, named Expressed Protein Ligation (EPL) [20,21]. In this case one or more of the peptide building blocks are prepared by recombinant DNA technology and ligated through a NCL reaction between a carboxy-terminal α-thioester and 1,2 aminothiol groups. For this reason, EPL is referred to as a semi-synthetic approach. To achieve C-terminal thioester or N-terminal Cys containing protein segments, EPL exploits a class of proteins called inteins, which are auto-cleavable elements able to catalyze their self-removal from flanking polypeptides [21,22]. EPL, as well as NCL, has been employed for the introduction into proteins of unnatural amino acids, post-translational modifications [6,20,23,24] and covalent dimers formation [25,26]. Furthermore, EPL, combining the advantages of organic chemistry with the easy and size limitless recombinant protein expression, allows for the preparation of modified large proteins.

### 1.2. Protein Synthesis via Intein Chemistry (EPL and PTS)

**Figure 4:**
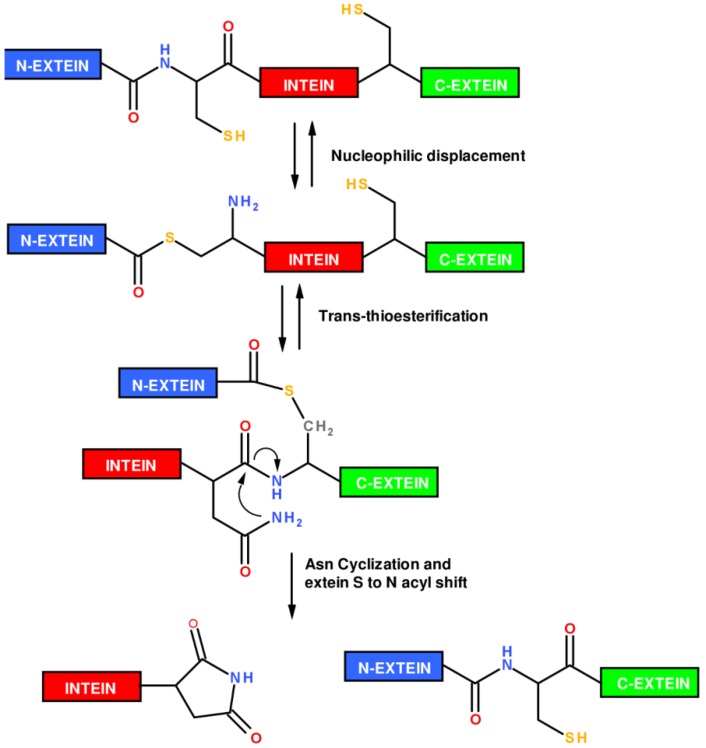
Mechanism of the intein protein splicing reaction.

The mechanism by which an intein is removed from a pre-mature polypeptide chain is known as protein splicing (Figure 4). Protein splicing proceeds through an N→S (or N→O) acyl shift in which the *N*-extein is transferred to the side chain -SH or -OH of a Cys/Ser residue, located at N-terminus of the intein. The N-extein is then transferred to a second Cys/Ser residue located at the N-terminus of the C-extein through a trans(thio)esterification reaction. A cyclization reaction involving a conserved Asn residue at the intein C-terminus leads to intein excision and to the formation of a new peptide bond between the two exteins, giving the mature polypeptide chain. A wide number of genetically engineered inteins able to catalyze their self-cleavage from only a N- or C-extein have been obtained, providing a powerful tool to prepare recombinant C-terminal thioester or N-terminal cysteinyl polypeptides required to perform EPL.

**Figure 5:**
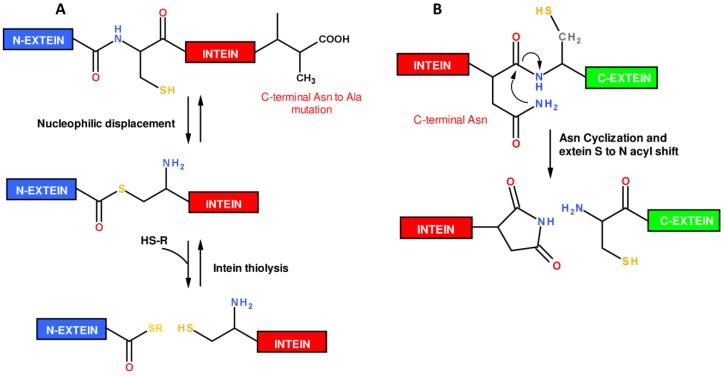
Schematic representation of reactions performed by engineered inteins yielding α-thioester proteins (**A**) or N-terminal cysteinyl-proteins (**B**).

To prepare C-terminal thioester proteins, inteins harboring the mutation of the C-terminal Asn into an Ala have been designed [22]. Such mutant inteins are unable to evolve from the thioester intermediate to the final spliced product. In this case, the thioester intermediate can be cleaved off by addition of thiols releasing the N-extein as C-terminal thioester protein (Figure 5A). Several *E. coli* expression vectors are commercially available that allow the recombinant expression of fusion constructs with engineered inteins, such as the *Saccharomyces cerevisiae* vacuolar ATPase subunit (*Sce* VMA) intein, *Methanobacterium thermo-autotrophicum* ribonucleoside diphosphate reductase (*Mth* RIR1) intein or the *Mycobacterium xenopi* DNA gyrase A (*Mxe* GyrA) intein [27]. The *Mxe* GyrA intein [28] is the most widely used as *Mxe* GyrA intein is small (198 amino acids), expresses at high level in bacteria, is able to efficiently refold from inclusion bodies and is cleaved by a variety of thiols, even in the presence of low concentration of denaturants (*i.e.*, 2 M urea), detergents or organic solvents [29]. Special care has to be devoted to the choice of the -1 residue at the junction extein-intein, as the efficiency of splicing is intein and N-extein sequence dependent. Furthermore, the extein sequence modulates the kinetic of protein splicing. Notably, a -1 Asp residue induces high levels of premature cleavage *in vivo*, whereas a -1 Pro should be avoided as completely inhibits the cleavage [20,27].

The thiol sodium 2-mercaptoethanesulfonate (MESNA) is often employed to mediate intein thiolysis. In fact, while thioalkyl-esters, obtained, for example, from ethanedithiol (EDT) and ethanethiol (ET), are quite stable, but not reactive enough in NCL, whereas thioaryl-esters, which result from the use of benzylmercaptan and thiophenol as splicing inducing agents, are much more reactive but even more susceptible to hydrolysis. Usually, an alkyl-thioester is preferred during the preparation and purification steps, while during NCL, the addition of aryl-thiols to the reaction mixture allows for conversion of the thioester species into a more reactive thioaryl-ester through trans-thioesterification. The thiol MESNA allows a good compromise between stability and reactivity of the thioester protein obtained. Furthermore, MESNA is a non-malodourous thiol, facilitating thioester protein handling and purification. A complete characterization of the reactivity of a wide series of thiols was performed by Johnson and Kent [30]. They also reported another useful non-maleodorous thiol, the *p*-mercaptophenylacetic acid (MPAA), which can be added to the NCL mixture to promote the *in-situ* generation of a very reactive thioester, resulting in a sensible improvement in the efficiency both in terms of yield and time of reaction.

Protein splicing can also be exploited to prepare amino-terminal Cys proteins. In this case, inteins have been mutated in order to induce the cleavage at the C-terminal splice junction, between the intein and a C-extein starting with Cys, through pH and temperature changes (Figure 5B). Splicing of such modified inteins allows for the release of an amino-terminal cysteinyl protein. The *Mxe* GyrA intein was also adapted to this application [27], as well as the *Synechocystis* sp. PCC6803 DnaB helicase (*Ssp* DnaB) intein [31]. A drawback of this intein-based approach refers to the spontaneous cleavage at the intein-extein junction which can occur during expression and purification of the fusion protein. Finally, due to their self-removing nature, inteins have been exploited as auto-cleavable fusion partner in protease-free purification schemes [32,33,34].

**Figure 6:**
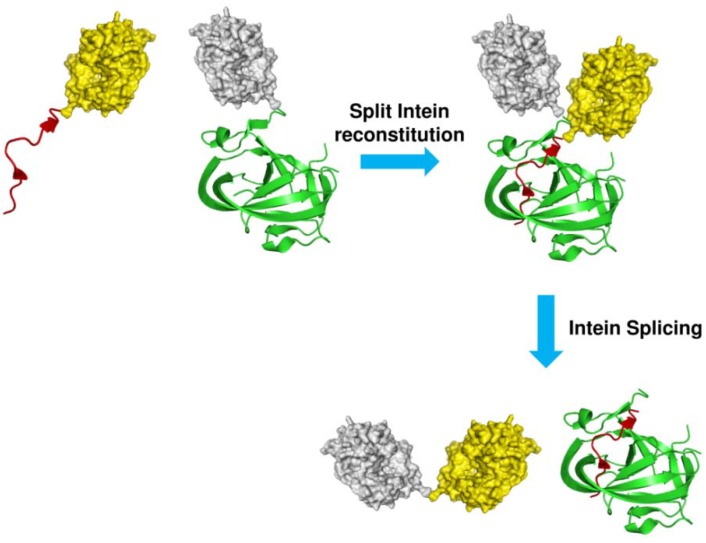
Schematic representation of the protein trans splicing reaction. Target proteins (yellow and gray) are fused to N- (IntN, red) and C-terminal (IntC, green) split intein fragments. After reconstitution, intein splices releasing the two target proteins linked through an amide bond.

Similarly to EPL, another process called Protein Trans-splicing (PTS) can be employed for the site-specific labeling of a protein. Such an approach relies on the use of a particular class of inteins which are naturally split or can be split into two pieces and, upon mixing, reassemble into a functional intein able to splice. Ligation of the proteins, which can be decorated with functional probes, fused to the split inteins occurs after the intein has been reconstituted (Figure 6) [35]. Natural or artificially split inteins have been examined to identify different possible sites of splitting. Interestingly, such investigations showed that in some inteins, such as *Mxe* GyrA, *Ssp* DnaB and *Ssp* GyrB inteins, the splitting site can be shifted very close to intein termini [36,37,38,39], rendering N-terminal or C-terminal split intein fragments so short that the preparation of this segments and their exteins can be accomplished by SPPS, expanding the repertoire of possible chemical modifications that can be introduced into a protein.

## 2. Fluorescent Labeling

Tagging proteins with fluorescent probes provides a tool to study proteins both *in vivo* and *in vitro* by confocal microscopy and fluorescence techniques, such as Förster resonance energy transfer (FRET), fluorescence correlation spectroscopy (FCS) and fluorescence polarization (FP). Genetic fusion to green fluorescent protein (GFP) and its color burst of spectral variants has allowed elucidation of the function of many proteins by mapping their intracellular localization and trafficking [40,41,42,43,44]. However, due to their large size, fluorescent fusion partners may interfere with protein folding and function. Moreover, fluorescent proteins quantum yield and photostability are quite limited [45]. Thus, it could be convenient to replace fluorescent partners with organic dyes [1,46]. Conventional methods for protein derivatization with fluorophores exploit the reactivity of native or engineered Lys or Cys side chains, as numerous dyes are commercially available as *N*-hydroxysuccinimide esters, isothiocyanates, iodoacetamides, vinyl sulfonones, maleimides and bromo-maleimides that react selectively with amino or thiol group [47,48]. However, this approach could be hampered by the presence of more than one Cys or Lys in the protein sequence, resulting in multiple labeling and heterogeneous protein preparations. Furthermore, many of such approaches suffer of a poor versatility, being tailored on a specific protein target [49,50]. Other approaches described are limited to the labeling of extra-protein sequence signal peptides, such as the reaction with FlASH, CrAsH or ReASH (biarsenical derivatives of fluorescein and resorufin) [51,52,53], which bind non-covalently to a short hexapeptide containing a tetracysteine motif (CCXXCC, were X stands for any amino acid, preferentially Pro or Gly), rhodamine-derived bisboronic acid (RhoBo) dye, which reacts with a tetraserine tag sequence (SSPGSS) [54], nickel-nitriloacetic acid derivatized chromophores [55,56], which interact with hexa or decahistidine tags, fluorophores complexed to Zn^2+^-2,2'-dipicoylamine based on L-tyrosine scaffold (Zn^2+^-DpaTyr), which bind to oligoaspartic acid sequence [57]. Enzymatic approaches for protein labeling were also described, such as the SNAP-tag [58], CLIP-tag [59] and Halo-tag [60] technologies. Other enzymatic strategies require the fusion of the target protein to a short signal peptide, such as the reaction catalyzed by sortase [61], transglutaminase [62], biotin-ligase [63], phosphopantetheine transferase [64], lipoic acid ligase, myristoyl-CoA:protein *N*-myristoyltransferase [65]. Although of great originality and inventiveness, the above mentioned labeling procedures are limited to N- or C-protein termini and generally do not allow incorporation of multiple, different molecular probes into a protein. The drawbacks of these fluorescent labeling strategies are successfully overcome using chemical ligation approaches, which do not require the addition to the protein of extra-sequence signal peptides or labeling domains and ensure open access to any site of any protein sequence with unique level of specificity.

### 2.1. Protein Labeling with Fluorescent Probes for FRET Studies

**Figure 7:**
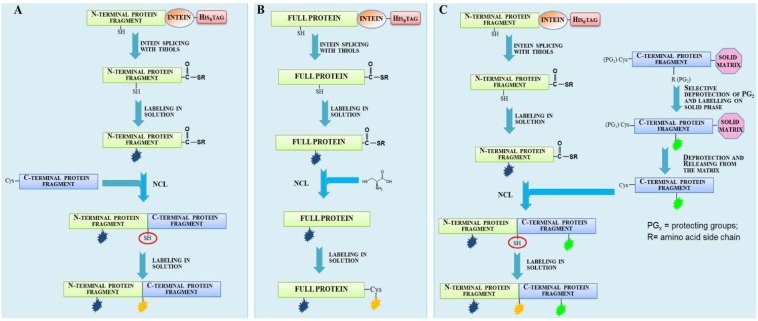
Schematic procedure to prepare double/triple modified proteins combining EPL and chemoselective labeling in solution [80].

A large number of fluorescent proteins have been prepared by EPL and employed for the conduction of structural and functional studies by FRET. FRET can be used to monitor dynamic processes involving protein structural changes, protein interaction or oligomerization through the variation of the spatial distance between two fluorophores. Relevant examples were reported by Muir, who pioneered the field with outstanding works [66,67,68,69]. Of great interest is his description of a solid-phase expressed protein ligation (SPPL) strategy that enabled the semi-synthesis of a large protein on a solid support. The designed approach was exploited to label the N and C termini of the Crk-II protein with the fluorescein and tetramethylrhodamine FRET pair. Crk-II was expressed in bacteria as fusion construct with the yeast vacuolar membrane ATPase (VMA) intein and with the affinity tag chitin binding domain (CBD). The fusion construct harbored an N-terminal Cys masked by the proteolysis site recognized by the factor Xa protease. The chimeric protein Xa site-Crk-II-intein-CBD was purified from cytosolic extract using a chitin affinity resin. The protein was incubated on the solid matrix with a synthetic peptide containing an N-terminal Cys residue and the probes fluorescein (Fl) and biotin, separated by the PreScission protease cleavage site (PS). The peptide reacted in an one pot reaction with the C-terminal thioester derivative of Crk-II protein, released from VMA intein splicing, affording the C-terminal Crk-II labeling with the first dye (Fl). The biotin handle was exploited to isolate the EPL product by an affinity chromatography step on an avidin-resin. Once bound to the resin, the Xa site-Crk-II-Fl PS biotin was incubated with factor Xa protease, in order to expose the N-terminal Cys while leaving the fusion construct attached to the resin. The protein underwent a second ligation reaction with a synthethic C-terminal-thioester peptide labeled with tetramethylrhodamine (Rd), affording the introduction of the second label. The doubly-labeled construct was cleaved from the resin upon incubation with biotin or with the PreScission protease. The latter method allowed the removing of the affinity biotin handle, giving the doubly-labeled species Rd-Crk-II-Fl. FRET experiments performed on dual labeled Crk-II construct before and after its phosphorylation by the nonreceptor protein tyrosine kinase c-Abl resulted in a variation of FRET efficiency between the dyes pair, suggesting conformational changes upon phosphorylation [67]. In a successive work, a doubly labeled truncated version of Crk-II that worked as fluorescent biosensor was synthesized, enabling real-time monitoring of c-Abl kinase activity and provided a rapid tool for screening potential c-Abl kinase inhibitors [68]. A combination of EPL and selective Cys-labeling was employed by the Ebright’s group to introduce the FRET pair tetramethylrhodamine and fluorescein into different subunits of the *E. coli* RNA polymerase holoenzyme [70,71]. FRET studies led to the elaboration of a structural model for the holoenzyme complex. More recently, Xie *et al.* used EPL to label the histone acetyltransferases (HATs) PCAF and p300 with Dabcyl as FRET acceptor, while HAT substrate analogues were labeled with the acceptor dye methoxycoumarin through SPPS. The molecules were subjected to FRET and fluorescence anisotropy assays to detect HATs inhibitors [72]. A FRET based approach to observe protein oligomerization was described by Scheibner *et al.* [73]*.* A synthetic dipeptide Cys-Lys(ε-fluorescein) (donor) or Cys-Lys(ε-rhodamine) (acceptor) was attached via EPL to the C-terminus of three recombinant proteins (glutathione S-transferase, SH2 domain phosphatase-1 and serotonin *N*-acetyltransferase) and a mixture of the two singly-labeled proteins, carrying the donor or the acceptor fluorophore, was analyzed by FRET. A similar labeling strategy was adopted in a study performed on two GTPases from the superfamily of Ras-like small GTPases, H-Ras and Ypt1, and two of their interacting partners, the Ras-binding domain (RBD) of c-Raf1 and MRS6. Target proteins were prepared as C-terminal thioester derivatives using intein fusion technology. Thioester proteins were reacted with synthetic decapeptides labeled in the solid phase with a flurophore and containing six His residues, the latter being useful to purify the ligation products. Labeled proteins were subjected to protein-protein and protein-nucleotide interaction studies by FRET and fluorescence cross-correlation spectroscopy (FCCS) [74]. Multicolor protein labeling is a useful way for the characterization of protein structure and folding by FRET studies. These studies require the attachment of a donor and an acceptor dye inside the same polypeptide chain at specific positions. An example of semi-synthetic protein doubly labeled with fluorescent dyes was reported by Yi *et al.* [75]. The described approach is limited to protein N- and C-termini and uses a combination of EPL and oxime ligation [76]. Rab7 GTPase protein was obtained as C-terminal thioester after intein splicing. The thioester protein was reacted with (bis)oxyamine moiety. The C-terminal oxamino-modified protein derivative selectively reacts with fluorophores containing a ketone functional group. To afford the second labeling, an N-terminal Cys residue was exposed by Tobacco Etch Virus (TEV) protease digestion of the protein which was then reacted by EPL with the second dye supplied as thioester derivative. The use of TEV protease to release an amino-terminal cysteinyl protein was introduced by Tolbert & Wong, who demonstrated that TEV protease could accept Cys in the P1 position of its proteolytic site rather than the usual Gly [77], enabling the use of TEV recognition site as “protecting group” for the N-terminal Cys of a recombinant protein in alternative to the factor Xa recognition site introduced by Muir [67]. In another example, Iakovenko and co-workers incorporated a fluorescent probe into a semisynthetic version of Rab7, a small GTPase [78]. The fluorophore was incorporated at the C-terminus of the protein, a region that is known to be post-translation prenylated by Rab geranylgeranyl transferase (RabGGTase). Using this approach, a library of 46 Rab7 analogs conjugated to different fluorophores was constructed and the molecules were used as sensors that report on the interaction of Rab7 with RabGGTase and the escort protein REP-1 [79]. Recently, we contributed to widen the applications of EPL describing a general method for the incorporation of two molecular probes at different, specific positions along the protein framework [80]. The approach is schematically illustrated in Figure 7 and combines protein semi-synthesis by EPL with the labeling in solution of a convenient functional group (a thiol in our case).

The protein of interest is split in two fragments (N- and C-terminal), possessing a single Cys residue. The N-terminal fragment fused to an intein is expressed in *E. coli* and spliced in presence of MESNA, affording the thioester derivative. Then the thioester protein is selectively labeled on its unique Cys residue, for example using a probe conjugated with the maleimide. Successively, the mono-labeled thioester fragment is reacted with an N-terminal Cys residue by a NCL reaction to afford full-length mono-labeled protein. Finally, the Cys residue involved in NCL reaction is exploited to introduce the second probe (Figure 7A). Unlike previously reported [81], both labeling reactions are performed in solution, enabling the use of mild excess of fluorophores and probes with a chemical structure that is not compatible with the harsh conditions of solid phase synthesis. A simpler version of our approach can be adopted when the second probe is located at the C-terminus (Figure 7B). In this case, the full-length protein containing a single Cys is prepared as thioester derivative and, after the first labeling in solution, is reacted with a Cys in an EPL reaction and then labeled with the second probe. As proof of concept, we successfully applied our synthetic approach to prepare four fluorescent variants of the repeat protein CTPR3 with different probes distances, confirming its efficacy and versatility. The protein variants were analyzed by CD and by ensemble-FRET. As the doubly-labeling strategy described requires RP-HPLC purification steps, the fluorescent proteins obtained are characterized by high purity and homogeneity, thus resulting amenable for single-molecule studies. This strategy could be also applied to prepare triple labeled proteins. In fact, the C-terminal fragment ending with a Cys residue could be chemically synthesized and selectively labeled on solid phase in a specific position. Then, it could be ligated to a labeled protein-thioester fragment to afford a doubly labeled species harboring a Cys residue exploitable for the introduction of the third probe (Figure 7C).

Semi-synthesis of fluorescent proteins can be also accomplished by PTS. Useful strategies have been described to tag the N- or C-terminus of a protein of interest with a chemical probe using specific split inteins characterized by a splitting site near to one end of the amino acid sequence, allowing for the preparation of the shortest domain of the intein by SPPS. For example, an engineered version of the *Ssp* DnaB intein was used by Ludwig *et al.* [38]. In this case the split site has been shifted at the amino acid 11, which is compatible with the chemical synthesis of the Int^N^ fragment and its extein. This approach was used to synthesize two fluorescein labeled proteins, the thioredoxin (Trx) and the β-lactamase (βLac). For the preparation of fluoresceinated Trx, the Int^C^ domain of *Ssp* DnaB intein (143 aa) was fused to Trx and an His_6_tag (Int^C^-Trx-His_6_). The construct was mixed in equimolar amount with a synthetic Int^N^ domain (11 aa) carrying a peptide labeled with a fluorescein as N-extein (Fl-extein-Int^N^). PTS yielded the fluorescent product Fl-Trx-His_6_ with a reaction rate of about 17 folds lower than that reported for *Ssp* DnaB intein split at previously known site, probably due to the strong impact that the new splitting site has on the reassembling and refolding attitude of the *Ssp* DnaB intein. Despite the reduced rate of splicing, the final trans-splicing yield (40–45%) was similar to those observed for *Ssp* DnaB intein split at the canonical site. The use of a higher excess of the synthetic domain did not result in an improvement of the final yield. The second protein used as case-of-study, βLac, required a refolding step, as the construct Int^C^-βLac-His_6_ aggregated in inclusion bodies during expression in prokaryotic hosts. Refolding was performed in one step by dialysis and the soluble Int^C^-βLac-His_6_ was reacted with Fl-extein-Int^N^, even in this case using equimolar amounts of the two reactants. PTS afforded Fl-βLac-His_6_ with a 35% yield. Both Fl-Trx-His_6_ and Fl-βLac-His_6_ were subjected to enzymatic activity assays, demonstrating that the semi-synthetic origin of the enzymes did not affect their functionality. The utility of the split *Ssp* DnaB intein has also been improved through the selection by a directed evolution approach of a mutant which shows a lower restriction about sequences requirements of the flanking exteins and an higher rate of trans-splicing [82]. Furthermore, a split variant of the *Ssp* GyrB intein with the split site near its C-terminus was designed, providing an N-terminal intein domain of 150 aa and a C-terminal intein domain of 6 aa only. The use of such intein allows for the chemical modification of proteins in the C-terminal region, in a similar fashion to that described to label a protein N-terminal region with *Ssp* DnaB intein. Protein *trans*-splicing has also been exploited to introduce a FRET pair into a single protein [83]. The reported strategy combines PTS on recombinant fragments and Cys-labeling in solution. The protein of interest was split at a Ser residue in two fragments (Prot^N^ and Prot^C^), each one bearing a single Cys residue along the amino acid sequence. Each fragment was expressed in fusion with a domain of *Npu* DnaE split intein (Int^N^ and Int^C^) and a solubility enhancing partners. The Ser residue at which the protein of interest has been split become placed at the catalytic junction between Int^C^-Prot^C^. Usually, inteins prefer a Cys residue at this site (however *Npu* DnaE intein tolerates also this nucleophile) allowing to realize the protein doubly-labeling in a three steps scheme. First, Int^C^-Prot^C^ construct was labeled at Cys with a thiol-reactive derivative of the first dye; the presence of a catalytic Ser instead of a Cys ensured to maintain intact the nucleophile at this position during Cys labeling. Then, the mono-labeled full protein was obtained through PTS by mixing Int^C^-Prot^C^ and Prot^N^-Int^N^. The second dye was finally introduced onto the full protein by labeling the only remaining Cys residue. As Int^N^ domain also contains a Cys residue involved in the splicing reaction, to preserve intein functionality, the labeling with the second dye was performed on the full protein after ligation instead than on the intermediate construct Prot^N^-Int^N^. The designed approach was validated through the synthesis of a doubly-labeled di-ubiquitin molecule, in which two ubiquitin units are joined by a linker sequence containing a Ser residue. Maltose binding protein was selected as enhancer of solubility and purification tag of the construct Int^C^-Ub while Ub-Int^N^ was expressed in fusion with a His_6_tag. Each unit of Ub bore a single Cys residue introduced by mutagenesis respectively at position 47 and 7. These sites were selected as they are exposed outside the Ub hydrophobic core. The doubly-labeled construct obtained was used to study the unfolding process by FRET. Recently, Muir’s group performed a systematic study of 18 cyanobacterial split DnaE inteins and several of them resulted to be “ultrafast” inteins, able to catalyze protein trans-splicing in tens of seconds, as previously observed for the *Npu* DnaE intein [84]. They analyzed the effect caused by C-extein sequence variation on ultrafast inteins trans-splicing rate, observing different degree of tolerance for each intein and kind of mutation. The ultrafast inteins appear an attractive and efficient tool to prepare α-thioester proteins for EPL [85].

The general applicability of PTS approach is however limited by the instability of the split protein fragments which can aggregate in inclusion bodies and for whom refolding procedures are not always successful. Besides, many inteins are not completely promiscuous regarding their exteins, showing often strong restrictions on the sequences that they tolerate in the proximity of the junction site extein-intein, limiting the number of protein targets that can be prepared by PTS.

### 2.2. Protein Labeling with Fluorescent Probes for Fluorescence Microscopy in Living Cells

The ability to track the position and the movements of proteins inside a living cell is a key approach to describe cellular mechanisms and protein functions. In the last years, we assisted to an explosion of chemical biology tools to label proteins for microscopy applications. Beck-Sickinger and coworkers described the site-specific fluorescent modification by EPL of the SDF1α chemokine. SDF1α (1–49) fragment was expressed as *Mxe* GyrA intein-fusion protein. Fusion protein was purified on chitin resin, exploiting the presence of the CBD at the intein C-terminus; thioester SDF1α (1–49) was obtained on resin after incubation with MESNA. The SDF1α (50–68) C-terminal peptide, starting with a native Cys, was synthesized by SPPS and labeled on solid phase with carboxyfluorescein at the C-terminal Lys residue. It was selected for labeling as it was assumed it did not interfere with the correct protein folding. EPL between the two fragments lasted 24 h. Since SDF1α contains two disulfide bridges, after ligation the full product was refolded and oxidized using the cysteine/cystine redox system. The well folded product was purified by HPLC, as the retention time of the oxidized species shifted with respect to the linear compound. Fluorescence microscopy studies carried out on the labeled chemokine demonstrated that the semi-synthetic SDF1α is biologically functional molecule, able to induce chemotaxis and to be internalized upon specific binding to its receptor CXCR4, supporting the extension of such synthetic protocol to other chemokine and to the introduction of other modifications [86]. EPL has also been harnessed to control the activity of a post-translational modified protein inside living cells by the use of photocleavable caging groups. In this approach, EPL is used to synthesize the protein of interest modified with a fluorophore and a caging group which quenches the fluorophore and, contemporary, suppresses the protein activity. The caging group is photocleavable and thus it can be removed upon light exposure ensuring to switch on protein activity and dye fluorescence emission. For example, this strategy was applied to the protein Smad2, involved in the transforming growth factor *α* (TGF-*α*) signaling pathway. The activation of Smad2 requires phosphorylation of two Ser residues placed at protein C-terminus. In the phopshorylated Smad2, the C-terminal carboxylate stabilizes a homotrimeric structure. The presence of a caging moiety at the C-terminus, able to disrupt the homotrimeric interactions, maintains the phosphorylated protein in an inactive state. UV irradiation induces the cleavage of the caging group and restores Smad2 activity. Smad2 analog harboring two phospho-Ser residues, a fluorescein as fluorescent probe, a dabcyl as quencher and the 4-[4-(1-hydroxyethyl)-2-methoxy-5-nitrophenoxy]butanoic acid as photo-cleavable caging group was prepared by semi-synthesis. Thioester Smad2 lacking the last five residues was prepared by intein-fusion technology, complexed with the membrane-anchored protein SARA and reacted with a synthetic peptide containing all the functional groups listed before [87]. In a subsequent work, the same concept and semi-synthetic strategy was applied to prepare caged phosphorylated and unphosphorylated Smad2, containing respectively the green fluorophore carboxyfluorescein and the red fluorophore tramethylrhodamine. Fluorescence appeared to be titratable by modulating the extent of UV-light exposition. The two proteins were co-injected into live cells and their fluorescence before and after photoactivation was monitored. This strategy enabled to track the phosphorylated and nonphosphorylated Smad2 inside the same single-living cell [88]. A C-terminal protein labeling was described by Chaisemartin *et al.* using a Cys analog linked to a N-1' fluorescent biotinyl derivative; the molecule was used to induce splicing of the fusion construct consisting of the intein *Mxe* GyrA and a scFv directed against the GTPase RhoB. Splicing afforded the fluorescent scFv which was purified to homogeneity exploiting the biotin hand [89], while fluorescent probe allowed the *in vivo* antibody detection. A “mirror” approach, useful to label protein N-terminus, has also been described and applied to the imaging in living cells [3]. In this work, the intein-mediated protein splicing allowed the *in viv*o generation of the target protein bearing an N-terminal Cys residue. EPL with membrane-permeant thioester-containing fluorophore allowed site-specific labeling of the protein. Other examples of N-terminal Cys containing proteins labeling with thioester molecules were also reported [90,91,92]. Protein labeling can be also accomplished *in vivo* by using protein trans-splicing, as firstly described by Giriat and Muir [93]. To validate their semi-synthetic approach, GFP was expressed in cells as fusion protein with the N-terminal fragment of *Ssp* DnaE split intein. The C-terminal *Ssp* DnaE intein fragment was instead fused to the FLAG epitope and supplied to the cell media. The latter construct was able to penetrate through cell wall thanks to a Protein Transduction Domain (PTD) peptide which served as a signal peptide and which was ligated to Int^C^-FLAG through a disulfide bridge. Western-blot analysis of the cell lysate with anti-GFP and anti-FLAG antibodies demonstrated the ability of the intein to reconstitute *in vivo* and splice giving the GFP-FLAG as product. The naturally occurring split intein *Npu* DnaE, characterized by the highest rate of trans-splicing reaction, has also been adopted to ligate an exogenous polypeptide to a membrane protein exposed by living cells, offering a useful way to modify a cell surface protein. The 36 amino acids C-terminal domain of the *Npu* DnaE intein (Int^C^) was expressed in eukaryotic cells as fusion with a transmembrane domain derived from PDGF receptor (TM), a soluble partner (Trx) and the fluorescent protein mCherry. Microscopy analysis on living cells showed that the construct Int^C^-Trx-TM-mCherry was localized on plasma membrane. Cells were incubated with a green fluorescent protein carrying the complementary moiety of the *Npu* DnaE intein (102 amino acids), the construct eGFP-Int^N^, which was expressed in *E. coli* and purified. Specific co-localization of mCherry and eGFP proteins at cell surface was verified by confocal microscopy, demonstrating that *Npu* DnaE intein was able to reconstitute into a functional splicing unit in a cellular context. *Npu* DnaE intein has two catalytic cysteines involved in PTS reaction that must be reduced to ensure successful trans-splicing. Authors observed that PTS was not compromised if the contruct eGFP-Int^N^ was preincubated with DTT and dyalized against PBS before the incubation with cells. Direct incubation of cells with DTT should be avoided as thiols may damage cell membranes. The semi-synthetic approach was extended to the preparation of proteins attached to the membrane through a glycosylphosphatidylinositol (GPI) anchor. To this scope, the Int^C^ domain was expressed endogenously in fusion with the GPI signaling sequence Gas1p, which triggers its attachment to a glycolipid anchor on the cell membrane. Incubation with eGFP-Int^N^ allowed membrane labeling through a GPI-anchor [94].

## 3. Isotopic Labeling

**Figure 8:**
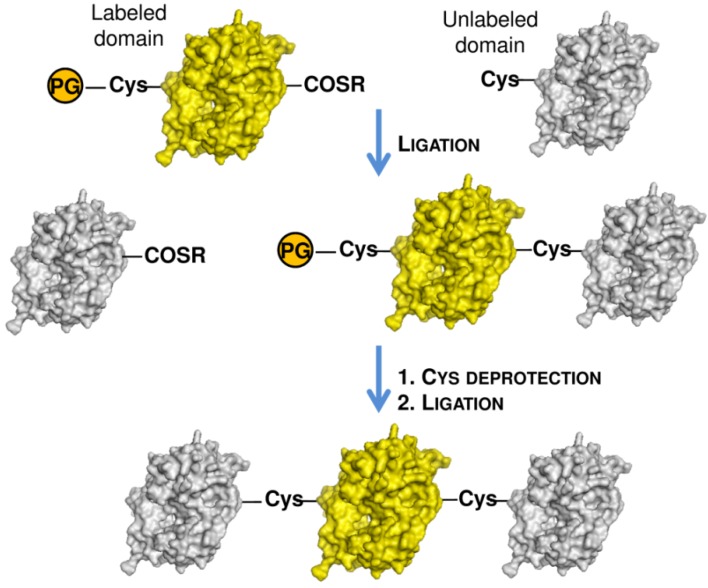
Semi-synthetic strategy for isotope labeling of an internal protein domain. The labeled protein domain is reported in yellow. PG is a protease recognition site which releases a N-terminal cysteinyl-protein.

Structural characterization by NMR spectroscopy may be not trivial if applied to large proteins, due to significant loss of spectral resolution and chemical shifts overlap. Both effects are progressively amplified as the protein size increases, hampering unambiguous signals assignment. The synthesis of large proteins by ligation methods, such as EPL, may resolve this issue. Through a synthetic approach, named segmental isotopic labeling, a selected portion of a protein, such as a single protein domain or region, can be specifically labeled with ^13^C, ^15^N and/or ^2^H isotopes and analyzed by NMR spectroscopy in the context of the native full protein (Figure 8). Segmental labeling allows to reduce the complexity of the NMR spectrum as only the signals of the labeled region are revealed, facilitating the structural analysis of the labeled protein portion.

Muir’s group reported the first example of segmental labeling by EPL [95,96]. In this work, EPL was used to prepare the Abelson protein tyrosine kinase-SH(3,2) domain pair, in which only one of the two domains was labeled with ^15^N. Unlabeled Abl-SH3 domain was prepared as C-terminal ethyl thioester using intein technology and reacted with N-terminal Cys containing (^15^N)Abl-SH2. A combination of mass spectrometry and NMR spectroscopy techniques was used to confirm the identity and the correct structure of the ligation product, which also showed the appropriate ligand-binding properties. The same semi-synthetic procedure was adopted by Camarero *et al.* to isotopically label the C-terminal region of a sigma70-like subunit from *Thermotoga maritima* in order to study the mechanism of autoinhibition mediated by its own N-terminal 90 amino acids by NMR [97]. More recently, the Gierasch’s group described the segmental isotopic labeling of the *E. coli* Hsp70 molecular chaperone DnaK [98]. One of its two constituent domains, the ATPase domain, was expressed as N-terminal fusion with the *Mxe* GyrA intein in minimal medium containing ^2^H, ^13^C and ^15^N isotopes sources, generating a labeled C-terminal thioester derivative after incubation with the thiol MESNA. The complementary substrate-binding domain (SBD) was expressed as C-terminal fusion with *Ssp* DnaB intein to generate, after intein splicing, a N-terminal Cys residue. The two domains were then ligated, affording full DnaK bearing the ATPase domain labeled with NMR active isotopes. In order to optimize ligation yield, many conditions were tested, evaluating the effects of varying pH, temperature, reagents concentration and ratio, denaturant concentration. Interestingly, they found appropriate to carry out the ligation reaction at low urea concentration (2 M) to enhance ligation efficiency, while higher or lower denaturant concentration resulted in a decreased ligation yield. They interpreted these results hypothesizing that urea concentrations higher than 2 M disrupt specific domain-domain interactions which bring together the ligands and promote ligation. At lower urea concentrations, instead, domains interaction persists but ligation yield falls down due to poor accessibility of C-terminal thioester group and N-Cys of the two domains. The former effect does not affect ligation yield if the reaction is carried out using millimolar reagent concentrations and an excess of SBD domain.

**Figure 9:**
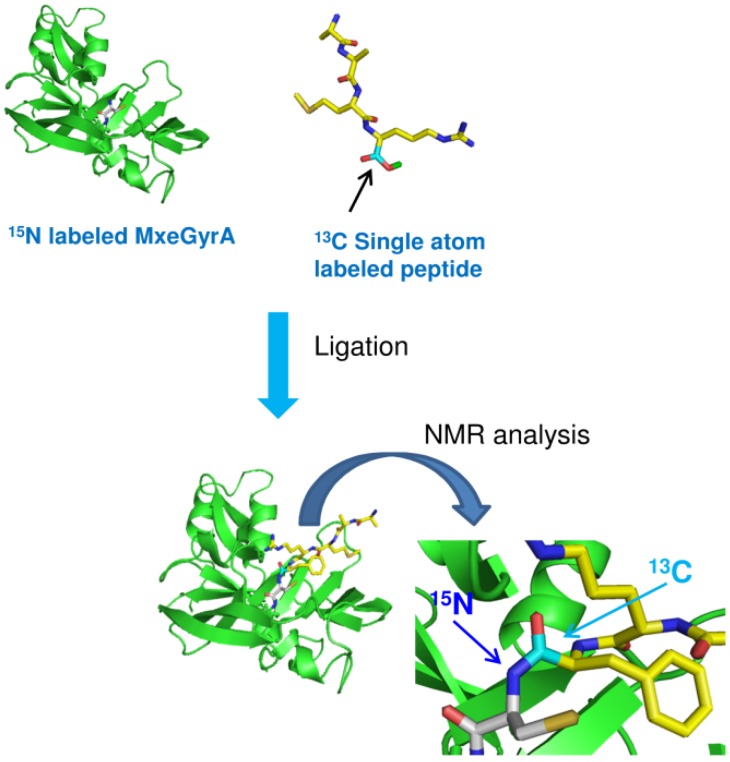
Semi-synthesis of *Mxe* GyrA intein with the scissile (-1) peptide bond dual labeled with ^13^C and ^15^N. *Mxe* GyrA intein (green) was uniformely labeled with ^15^N. Extein peptide (yellow) was synthesized by SPPS with a ^13^C α-carboxylate.

Vitali *et al.* described an interesting segmental labeling reaction performed on resin, which requires the concerted use of two inteins, the *Mxe* GyrA and *Ssp* DnaB inteins. The synthetic procedure was adopted to prepare and characterize by NMR a series of segmentally labeled variants of the RNA recognition motifs (RRM3 and RRM4) of the polypyrimidine tract binding protein (PTB). The RRM3 motif was expressed in fusion with *Mxe* GyrA intein and a CBD as affinity partner, while RRM4 was fused at the C-terminus of the *Ssp* DnaB intein and at the N-terminus to a CBD. As both fusion proteins bore the same affinity tag, they were co-loaded onto a chitin resin and the splicing of each intein was induced. Firstly, *Mxe* GyrA intein was spliced upon incubation with MESNA, releasing a C-terminal α-thioester RRM3. Then, a temperature switch to 37 °C allowed *Ssp* DnaB intein splicing, leaving RRM4 domain with an amino-terminal Cys residue. Once released, the two domains promptly reacted, affording a unique polypeptide chain. The full-length product was finally purified on Ni^2+^-resin, exploiting the His_6_ tag placed upstream RRM3 as additional affinity moiety. The ability of the two domains to interact facilitated their successful ligation. Segmental labeling could simply be accomplished loading onto the chitin resin an isotopically labeled RRM-Intein-CBD construct. By the use of the described on column procedure, many segmentally-labeled variants of RRM34 were prepared carrying ^15^N,^13^C-labeled RRM3 and an unlabeled RRM4, unlabeled RRM3 and a ^15^N,^13^C-labeled RRM4 and a ^15^N,^13^C-labeled RRM3 and a ^15^N-only-labeled RRM4 [99]. The same procedure was adopted and improved by Skrisovska and Allain, who described the characterization of two different multidomain proteins containing RNA recognition motifs (RRMs), heterogeneous nuclear ribonucleoprotein L and Npl3p. In this work, the splicing-ligation on column procedure was extended to insoluble protein, adding a refolding step before protein binding on resin, and to non-interacting domains, for which full-length protein yield was enhanced by eluting from the resin and concentrating the non-ligated reactants. The proteins were obtained in high yields, allowing to characterize their interdomain interactions by NMR spectroscopy [100]. An elegant approach was also proposed by Zhao *et al.* that reported an efficient on-column EPL strategy for the semi-synthesis of human apolipoprotein E (apoE) triply-labeled with ^2^H, ^15^N and ^13^C. The protein was expressed in two fragments. The N-terminal portion of ApoE was expressed as intein fusion construct and also bore a CBD as affinity tag. After binding on a chitin-resin, the fusion protein was incubated with the C-terminal ApoE fragment, also prepared by recombinant means and harboring a N-terminal Cys, to perform on-resin ligation reaction. Using this on-column ligation approach, once generated from intein thiolysis, the thioester species may readily react with the N-terminal Cys-fragment, strongly reducing the probability of hydrolysis of the thioester group and increasing the ligation yield. By this protocol, several variants of ApoE were prepared combining the two fragments labeled with three different NMR active nuclei, ^2^H, ^15^N or ^13^C [101,102]. Another biological problem which was addressed by segmental labeling is the study of ubiquitin biology. The attachment of a ubiquitin (Ub) or of poly-Ub to a target protein may regulate a great number of cellular processes, such as protein proteasomal degradation, transcriptional activation, vesicular trafficking of proteins. Poly-Ub chains are made by linking to each other Ub monomers through an isopeptide bond between the ε-amino group of one of the seven Lys residues of a monomer with the C-terminus of the next one or head-to-tail. Depending on the Lys involved into poly-Ub assembling, poly-Ub chains may work as molecular signals for different biological processes. Probably, the specificity of each poly-Ub chains depends on the different conformation that each one assumes depending on the sites of ramification. The characterization of the interaction of different poly-Ub with their receptors requires the ability to prepare homogeneous preparation of poly-Ub with a defined linkage between each Ub monomer. EPL fulfills such request, being more selective and versatile than enzymatic methods. However, due to its homo-polymeric nature, NMR studies of poly-Ub chains represent a challenging task. Segmental labeling offers a solution to the problem, allowing to observe each single Ub monomer in the context of the branched poly-Ub. Castaneda *et al.* reported the semi-synthesis of a segmentally isotopic labeled Ub_2_ chains. A Ub monomer carrying a δ-mercaptolysine at two positions (33 or 48) was prepared by chemical synthesis and reacted with a C-terminal thioester (^15^N)-labeled Ub monomer, obtained by recombinant expression as intein fusion protein. EPL allowed the formation of an isopeptide bond between the two monomers. After ligation, the thiol group in δ-position of the Lys was removed by desulfurization, affording a native- like di-Ub molecule [103]. The variant in which the two Ub monomers are linked through Lys48 was used as control construct, as this poly-Ub has been widely characterized by NMR. Data obtained using the segmentally labeled synthetic K48-linked di-Ub demonstrated that it is structurally identical to the one assembled using enzyme, confirming that the protein chains obtained by EPL are ‘‘native-like’’ and paving the way to the structural, conformational and ligand-binding properties characterization by NMR of the K33-linked di-Ub variant. EPL is a useful approach even for the site-specific introduction of one or few stable isotopes within a protein. Romanelli *et al.* applied the segmental isotopic labeling to selectively dual label the scissile peptide bond at the N-extein/intein junction of *Mxe* GyrA intein with ^13^C and ^15^N nuclei. ^15^N uniformly labeled *Mxe* GyrA intein was ligated to a synthetic pentapeptide labeled with ^13^C only on the C-terminal carboxylic group (Figure 9). On such construct they were able to measure the amide ^1^J_NC’_ coupling constant which was found to be of 12.3 Hz. This result suggests that this amide bond is unusually polarized, because of non-planarity, allowing one to explain the extreme lability of the N-extein-intein bond which is broken in the first step of protein splicing. Additional studies demonstrated that a conserved His residue of intein block B is absolutely required to catalyze the first step of the splicing reaction and contributes to maintain the (-1) scissile bond in its unusual conformation [104]. Further investigations on *Mxe* GyrA intein splicing mechanism were carried out performing NMR studies on a semi-synthetic branched intermediate prepared ligating a recombinant 1–184 α-thioester intein fragment with a synthetic branched peptide reproducing the intein remaining portion (aa 185–198) with the C-extein and carrying the N-extein attached to the side chain of a Thr. Furthermore, the ^13^C and ^15^N isotopes were specifically incorporated into the scissile +1 peptide bond [105]. In another example, a series of ^13^C-labeled amino acids were incorporated at the C-terminus of the α-subunit of a heterotrimeric G protein [106]. Using EPL, 9-mer peptides containing ^13^C labels in Leu-348 (uniform), Gly-352 (alpha carbon), and Phe-354 (ring) were ligated to recombinant Gα subunit lacking the corresponding carboxyl-terminal residues. Analysis of the ^13^C resonances indicated that the C-terminus of the Gα subunit is unstructured when the protein is bound to GDP, but adopts an ordered conformation upon activation by AlF_4_^−^.

EPL allows even for the segmental labeling of a protein internal region. To this purpose EPL makes use of sequential-ligation tools, as firstly described by Muir’s group who semi-synthesized the 304 amino acids Crk-II protein through the sequential assembling of three recombinant polypeptides. In such approach, the central segment bears both a C-terminal thioester and a “cryptic” N-terminal Cys masked by a protease cleavage site which prevents the self-reaction of the central polypeptide. After EPL between the central thioester segment and the C-terminal polypeptide, protease cleavage allows to reveal the α-Cys in the intermediate protein. Reaction with N-terminal segment, harboring the C-terminal thioester function, may afford the full protein [107]. Zaragöz *et al.* applied EPL to the semi-synthesis of the dimeric protein Hsp90, evaluating the effect on ligation yield of varying critic parameters such as reactants concentration, pH, ligation site. They developed an EPL based protocol suitable for the preparation of segmentally labeled dimeric proteins [108]. Another application of segmental labeling refers to infrared spectroscopy. Moran *et al.* applied segmental labeling to the study of human γD-crystallin amyloid fibrils by two-dimensional IR spectroscopy. EPL was used to uniformly label with ^13^C one of the two Greek key domains in order to individually resolve them in (2D) IR spectra of acid-induced amyloid fibrils [109,110]. Segmental labeling can also be accomplished by intein trans-splicing. This approach has been, recently, exploited by the Iwai’s group which described a strategy to label an internal region of a protein of interest using the split intein *Npu* DnaE [111] and more recently the preparation of a segmentally labeled version of the multi-domain CheA protein from *E. coli* both by EPL and trans-splicing [112]. In the latter work, pTWIN vector was used to express the two domains of the 140 kDa protein CheA respectively as *Mxe* GyrA and *Ssp* DnaB fusion constructs. EPL between the two domains was performed in native conditions and thus gave a ligation yield of only 10%–40% due to the impossibility to reach high reactant concentrations under non-denaturing conditions. PTS performed using *Npu* DnaE intein gave better results because the trans-splicing reaction does not require high reactant concentrations as the two intein fragments show a very high affinity. Intein trans-splicing mediated segmental labeling was also applied to study F_1_-ATPase, whose characterization by NMR is hampered by its amino acid length. A segmental labeling procedure based on the use of PI-*pfu*I intein was applied to label the β subunit of the protein and allowed to successfully assign the great majority of signals of the beta subunit monomer. The structures elaborated from the collected NMR data suggested that the subunit beta monomer assumes the open form in the absence of the nucleotide, while nucleotide binding induces a conformational change from the open to the closed form. The structural change of the beta subunit monomer induced by nucleotide binding triggers the rotation of F_1_-ATPase [113]. In a subsequent work, the segmentally labeled F1-ATPase was studied to gain deeper insights into the rotation of F1-ATPase, driven by the open/close bending motion of the β subunit [114]. An interesting approach has been described by the Iwai’s group to segmentally label a protein by *in vivo* PTS [115,116,117]. In this synthetic scheme the two protein fragments of a target protein are expressed in fusion with the N or C domain of a split intein. Each fusion construct is placed under the control of an inducible promoter, for instance the T7 and arabinose promoters respectively inducible by IPTG and arabinose. Bacterial cells co-transformed with both the recombinant plasmids are cultured and subjected to two sequential induction steps. First, labeled domain expression is induced in minimal medium containing isotopes source; then, cells are harvested by centrifugation, resuspended in rich culture medium and then expression of the complementary domain is induced. *In vivo* PTS affords segmentally labeled full-length protein target. Such approach has been used by Iwai’s group to prepare a ^15^N-segmentally labeled variant of the c-CRKII adaptor domain using the split intein *Npu* Dna E [115]. Segmental labeling by PTS has also been adopted to overcome the solubility limit of a protein sample for NMR studies. In fact, a common obstacle to NMR studies of proteins is the preparation of samples in soluble form at high concentration, usually in the millimolar range. To improve protein solubility, fusion of the target protein to a solubility enhancement tag (SET), such as the glutathione S-transferase, the maltose-binding protein or the thioredoxin, allows one to overcome the problem and at the same time facilitates the purification procedures. However, many extra signals arise from the SET, complicating the NMR spectrum. Segmental isotopic labeling offers the right solution to the quest trough the so called “invisible SET’’ approach, referring to the preparation of a SET fusion protein in which solely the target protein is isotopes enriched. Using *in vivo* intein trans-splicing, the non-isotope labeled SET protein GB1 (*Streptococcus* Protein G B1 domain) was fused to ^15^N-labeled chitin binding domain (CBD), improving its solubility for a study by solution NMR spectroscopy [117]. Although of great originality, the shortcoming of such approach is that the target protein does not possess a stabilizing fusion partner during expression and thus it may aggregate into inclusion bodies before *in vivo* ligation, becoming unable to react with the SET. In order to overcome such limitation, Kobayashi *et al.* developed an alternative invisible SET approach, always based on intein trans-splicing mechanism. The target protein, the ribosome binding factor A (RbfA), was expressed in isotopes enriched medium as fusion construct with a tandem repeated N-terminal domains of the protein S from *Myxococcus xanthus*, previously reported to be a useful SET [118], and with the C-terminal domain of a split intein. The same SET protein was expressed fused to the split N-terminal domain of the intein. Intein trans-splicing between them allowed to swap the labeled SET from RbfA with an unlabeled one. Using this approach, RbfA soluble expression is ensured by fusion with protein S tag even during expression phase and NMR characterization is facilitated by suppressing SET signal trough segmental labeling [119].

## 4. Conclusions

Protein semi-synthesis by EPL is nowadays a mature technology, it has demonstrated its utility to solve numerous scientific issues, especially in chemical biology and biophysics. In particular, EPL is one of the favorite methods to prepare high pure and homogenous proteins modified with specific probes, as fluorescent dyes or isotopes. This aim is achieved developing several ingenious synthetic strategies, indicative of the flexibility of EPL technology. In fact, EPL combines the advantages of organic chemistry with the easy and size limitless of recombinant protein expression, enabling a single protein to be functionalized at one or even more specific positions with different probes irrespective of its size. In definitive, EPL is an excellent strategy for the chemical synthesis of labeled protein which can find application in wide variety of life-science laboratories.
